# Relation of Non-Suicidal Self-Harm to Emotion Regulation and Alexithymia in Sexually Abused Children and Adolescents

**DOI:** 10.1192/j.eurpsy.2023.1342

**Published:** 2023-07-19

**Authors:** M. M. Mohamed

**Affiliations:** psychiatry, ain shams faculty of medicine, cairo, Egypt

## Abstract

**Introduction:**

Globally, children are abused sexually. It physically and mentally strains society. Abusers can develop eating problems and non-suicidal self-harm. Emotion regulation links purging, NSSI, and abusive situations. We examined 80 13-20-year-olds, 62.5% of whom had CSA, and 30 healthy controls. Victims were given the Toronto Alexithymia Scale, an eating disorders clinical interview, the Difficulties in Emotion Regulation Scale to assess emotion dysregulation, the Self-punishment Scale to assess NSSI, the Mini-Kid for children under 18 and the Structured Clinical Interview for DSM-IV Axis I Disorders (SCID I) for those 18 and older. 62.5 percent have CSA. CSA was connected to emotional dysregulation. Alexithymia is connected with problems describing and identifying feelings and outside oriented thinking. CSA patients exhibited severe self-punishment symptoms, greater than controls. Kids and teens often have CSA.

**Objectives:**

to look into the link between CSA and NSSI, as well as Alexithymia, emotional eating, and emotion dysregulation.

**Methods:**

We interviewed 80 mental outpatients from October to February 2019. 30% of healthy controls have CSA. Participants were 10–24-year-olds without PTSD or ASD. Mini-Kid is a 10- to 18-year-old neuropsychiatric interview (Sheehan et al., 1998), Self-injury scale measures non-suicidal self-harm (NSSI), Problems (DERS; Bjureberg et al., 2016) TORONTO ALEXITHYMIA QUESTIONNAIRE (Bagby et al., 1994). The Eating disorders clinical interview (Kutlesic et al., 1998)

**Results:**

**Table.** Describing the difference between control group and patients’ group regarding self- punishment scale.

	Control group	Patient group		
Self-punishment scale		N (%)	N (%)	P value
Physical punishment	Mild	18 (60)	9 (18)	<0.001
	Moderate	11 (36.7)	28 (56)	
	Severe	1 (3.3)	13 (26)	
Thinking & affective punishment	Mild	15 (50)	9 (18)	0.001
	Moderate	11 (36.7)	16 (32)	
	Severe	4 (13.3)	25 (50)

**Conclusions:**

CSA survivors had higher rates of self-injury, emotional eating, alexithymia, and emotional dysregulation than healthy controls. CSA victims should be evaluated for non-self-injury, emotional dysregulation, and emotional eating.

**Disclosure of Interest:**

None Declared

